# Cranial and Odontological Methods for Sex Estimation—A Scoping Review

**DOI:** 10.3390/medicina58091273

**Published:** 2022-09-14

**Authors:** Laura Maria Beschiu, Lavinia Cosmina Ardelean, Codruta Victoria Tigmeanu, Laura-Cristina Rusu

**Affiliations:** 1 Department of Oral Pathology, Multidisciplinary Center for Research, Evaluation, Diagnosis and Therapies in Oral Medicine, “Victor Babes” University of Medicine and Pharmacy Timisoara, 2 Eftimie Murgu Sq., 300041 Timisoara, Romania; 2 Department of Technology of Materials and Devices in Dental Medicine, Multidisciplinary Center for Research, Evaluation, Diagnosis and Therapies in Oral Medicine, “Victor Babes” University of Medicine and Pharmacy Timisoara, 2 Eftimie Murgu Sq., 300041 Timisoara, Romania

**Keywords:** sex estimation, cranial methods, odontological methods, morphometric analysis, morphologic analysis, biochemical analysis

## Abstract

The estimation of sex from osteological and dental records has long been an interdisciplinary field of dentistry, forensic medicine and anthropology alike, as it concerns all the above mentioned specialties. The aim of this article is to review the current literature regarding methods used for sex estimation based on the skull and the teeth, covering articles published between January 2015 and July 2022. New methods and new approaches to old methods are constantly emerging in this field, therefore resulting in the need to summarize the large amount of data available. Morphometric, morphologic and biochemical analysis were reviewed in living populations, autopsy cases and archaeological records. The cranial and odontological sex estimation methods are highly population-specific and there is a great need for these methods to be applied to and verified on more populations. Except for DNA analysis, which has a prediction accuracy of 100%, there is no other single method that can achieve such accuracy in predicting sex from cranial or odontological records.

## 1. Introduction

The estimation of sex from osteological and dental records has long been an interdisciplinary field of dentistry, forensic medicine and anthropology alike, as it concerns all the above mentioned specialties.

In both forensic and archaeological cases, a reliable method to establish the sex of the deceased is paramount, as it is the first step towards a more detailed analysis of the human remains and helps in narrowing down the list of individuals and putting together a demographic pattern.

**Figure 1:**
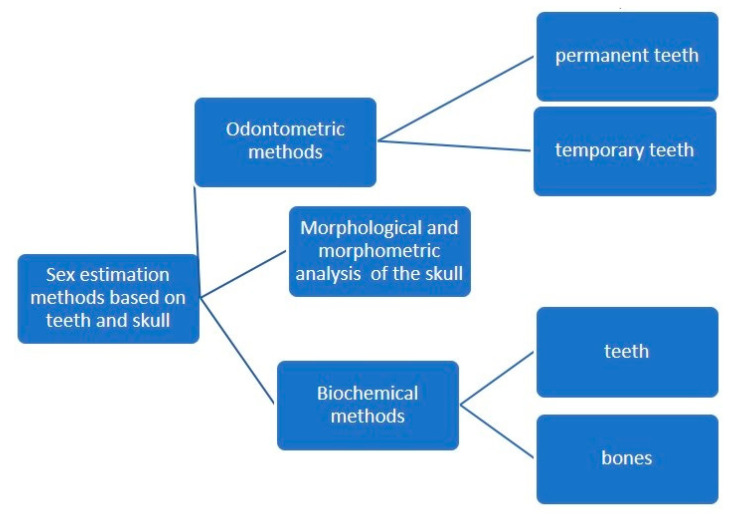
Odontological and cranial sex estimation methods (overview).

The estimation of sex from osteological remains can be achieved using three major types of methods: morphological assessment (non-metric) of teeth and bone traits that exhibit dimorphic features, morphometric assessment (by measuring specific quantifiable features of bones and teeth) and biochemical analysis, such as DNA analysis [1,2,3,4] or Barr bodies analysis [5] (Figure 1). DNA analysis is by far the most accurate method, but it is also the most expensive and may not be suited for large numbers of specimens [6,7].

### 1.1. Morphological and Morphometric Methods

The morphological and morphometric assessment methods are both generally accepted techniques based on scientifically proven grounds, but they have limitations. For instance, morphological assessment (non-metric) is based on a certain subjective evaluation of the observer and also requires experience. Morphometric assessment (metric), on the other hand, is a laborious technique and depends on the exact determination of anatomical landmarks. Moreover, the population-specific variations in the skull make these methods almost impossible to generalize [8].

More recently, computer-aided techniques have facilitated the use of morphometric assessments, making them less subjective and time-consuming. Advances in three-dimensional image analysis have achieved rapid, automatic measurement of the entire outer surface of the craniofacial hard and soft tissue, as opposed to measurements of only limited distances and angles of the cranium. The digital analysis of the cranium and digital data storage have had a huge impact on sex estimation methods. The stored images, whether digital impressions or radiographic images, can be used time and time again for multiple analyses [9,10,11].

Almost all bones exhibit dimorphic features. Sex discrimination methods have proven successful in many bones, including the hyoid, ulna, sternal end of the rib, metacarpals and even metatarsals [12]. However, the pelvis shows the highest degree of dimorphism, followed by the skull [13], which has an accuracy for gender determination of up to 94% [14].

**Figure 2:**
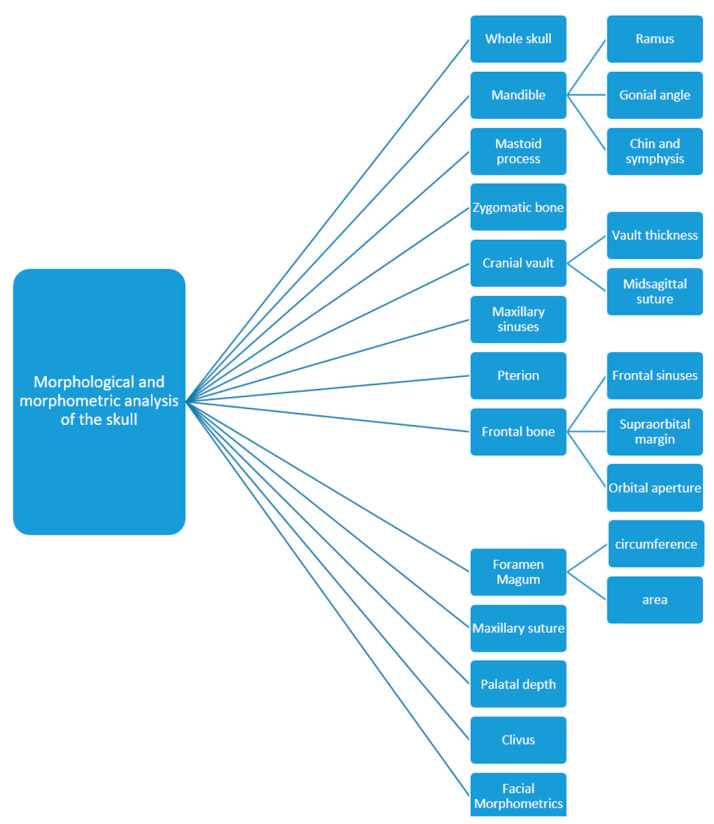
Parts of the skull used for sex estimation.

The anatomical structures of the skull used for the purpose of sex estimation are numerous: the frontal bone (position of squamous part, the appearance of the supraciliary arch, the sharpness and shape of the orbit, the frontal sinus—which remains stable and unchanged until old age and is, according to some studies, a unique structure, comparable to fingerprints) [15], the zygomatic bone (presence of marginal tubercle on the frontal process), the temporal bone (size and shape of the mastoid process, width of the zygomatic processes), the occipital bone (the nucal crest, the clivus), the mandible (angle between body and mandible ramus—angle of mandible, ramus height, base height), the shape of the nasal root, muscular insertions on bones, tooth size, face shape etc. [16] (Figure 2).

In many circumstances, whether in mass fatalities, explosions, mutilated bodies or poorly preserved archaeological records, the entire pelvis or skull cannot be retrieved and only fragmented parts of these bones are available for study. In these cases, the mandible plays a decisive role in sex estimation because it is the largest, strongest and one of the most dimorphic parts of the skull [17,18,19]. Dimorphism in the mandible is reflected in its shape and size; male bones are generally bigger and more robust than female bones. If only the mandible is available for assessment, gender determination has an accuracy of around 90% [16].

The mandible is usually one of the best preserved bones, along with the teeth, which are highly resistant to bacterial degradation, extreme heat and other types of aggressions and are therefore most likely to be preserved in fossil and archaeological records. Teeth can be heated to temperatures of 1600 °C without appreciable loss of microstructure [20] and, unlike skeletal bones, the human origin of teeth is rarely in doubt [21]. That is why the teeth form a highly valuable asset in estimating the sex of deceased individuals and are especially important in assessing children, where dimorphic aspects of the pelvis and other bones are not yet recognizable. In cases of fire or explosion, the thermal trauma causes major damage to the anatomical structures, leaving the teeth as the only way to establish the sex of the victims.

### 1.2. Biochemical Methods

Biochemical analyses for sex estimation purposes re based on DNA and Barr bodies from the dental pulp or from the hard tissue of the teeth. The DNA polymerase chain reaction (PCR) is more expensive and takes longer to obtain results, whereas the Barr bodies analysis is quicker and requires less equipment [5,22].

Due to their great tissue resistance, teeth can be considered as a reliable source of DNA, making them valuable in biochemical analysis methods as well. All structures of the tooth have proven value for extracting DNA material (enamel, cementum, dentine and pulp). The dental pulp contains fibroblasts, odontoblasts, endothelial cells, peripheral nerves, undifferentiated mesenchymal cells and nucleated components of blood, found in the coronal and radicular pulp, which are rich sources of DNA and free from contamination by external factors [23].

Amelogenin (AMEL) is the enamel-specific matrix formed during the first stages of tooth formation. It has been discovered that there are two types of AMEL genes, one found on the X chromosome and the other found on the Y chromosome. Hence, using PCR on the AMEL gene from DNA found in the dental pulp is a useful method to establish the sex of an individual [23]. PCR analyses that target regions of the amelogenin gene have become the method of choice for sex estimation of biological samples [24]. However, discrepancies have been noted with AMEL gene-based sex estimation, mostly due to X and Y deletion in the population and mutations in primer-binding sites. Some populations, such as Indians, appear to be affected by high frequencies of Y deletion. The presence of PCR inhibitors, degradation of the DNA samples and the presence of mixed DNA also contribute to inaccurate results obtained by amelogenin analysis and, therefore, other alternative techniques and markers have been suggested for sex estimation, such as STS, SRY, TSPY, DXYS156, SNPs, DYZ1 and next generation sequencing (NGS) [25].

Among the methods used to extract DNA from the dental pulp, the method using phenol chloroform appears to be quite cost-effective, but it is tedious and requires high precision. Newer extraction methods, such as Chelex 100^TM^ (Medox Biotech, Chennai, India) and QIA cube^TM^ (Qiagen, Hilden, Germany), could be substituted for the traditional method [23]. Recently, another method, termed the loop-mediated amplification method (LAMP reaction), which can give results within an approximately half an hour time limit, has been recommended as an alternative to conventional PCR techniques. Another advantage of the LAMP method is that it works under isothermal conditions, which stops further denaturation of the DNA [24].

Other biochemical analysis methods include the use of a fluorescent body test. It has been shown that, when chromosomes are stained with quinacrine mustard, they fluoresce differentially along their length when viewed under ultraviolet light, and the human Y chromosome fluoresces more brightly than the other chromosomes [20]. The reason for the bright fluorescence of the Y chromosome is not entirely clear. This technique has been used in forensic science for sex estimation from dried blood stains, saliva and hair since the 1970s [20]. The fluorescent Y body test has shown to be a reliable, simple and cost-effective technique for gender determination in the immediate postmortem period of up to one month after death. Therefore, its limitation is related to the post-mortem interval, making it only relevant for recently deceased individuals and, hence, impossible to use in archaeological findings [20].

The estimation of sex in ancient archaeological remains and fossils is also possible through DNA extraction techniques. The dawn of ancient DNA (aDNA) techniques was in 1983 at Berkeley, California, when Higuchi et al. extracted and sequenced ancient mitochondrial DNA (mtDNA) from a 150-year-old specimen of the quagga, a zebra-like species [26]. Then, in 1985, Svante Pääbo successfully investigated 23 Egyptian mummies for DNA content [27] and, in 1997, aDNA from Neanderthal specimens from the Feldhofer Cave in Germany was also successfully extracted [28].

Even today, the retrieval of mtDNA from ancient human specimens is not always successful owing to DNA deterioration and contamination. Usually, only short DNA fragments can be retrieved from ancient specimens. Degradation and contamination in long-term preserved specimens still make analysis very difficult. This is due to the technical difficulties with extraction, amplification and sequencing of ancient mtDNA. In recent years, NGS has mainly been applied to ancient samples. It seems that this technique is suitable for aDNA research [29]. According to the literature, short tandem repeat (STR) typing could represent a time-saving and cost-effective solution for sex estimation in archaeological sites [30].

The aim of this article is to review the current literature regarding methods used for sex estimation based on the skull and the teeth, covering articles published between January 2015–July 2022.

## 2. Materials and Methods

A digital search of PubMed/Medline and DOAJ was performed using the following criteria: “sex” AND (“determination” OR “estimation” OR “prediction”) AND (“odontometric” OR “teeth”), “sex” AND (“determination” OR “estimation” OR “prediction”) AND “human skull”, “sex” AND “teeth” AND “ancient DNA“. Filtering of the publication period was applied. The search retrieved 832,715 results. These results were then refined by their title and abstract so as to be in accordance with the inclusion criteria. The reference list of all identified articles was further manually searched for additional articles. This process of refining and excluding eventually left a total number of 97 articles. The set question was: *What methods are used for cranial and odontological sex estimation and which ones have the highest prediction accuracy?*

The PICO specialized framework was used to form the question and facilitate the literature search.

- Population: all ages, genders and ethnicities included;
- Intervention: cranial and odontological methods for sex estimation;
- Comparison: age range, sample size, method used, sex estimation accuracy;
- Outcome: to determine the methods and find out which ones have the highest sex estimation accuracy.

The inclusion criteria comprised the following:

- Population included: all ethnic groups;
- Patients, autopsy cases and skeletons from archaeological records;
- Original articles;
- With or without abstracts;
- Articles written in English;
- Methodologies based on both skull and teeth assessments;
- Both metric and non-metric methods;
- Both temporary and permanent teeth;
- Study focus relevant to our search question;
- No minimum number of individuals required.

The exclusion criteria comprised the following:

- Studies covering non-human subjects;
- Studies published before 2015;
- Abstracts without full reports;
- Review articles.

**Figure 3:**
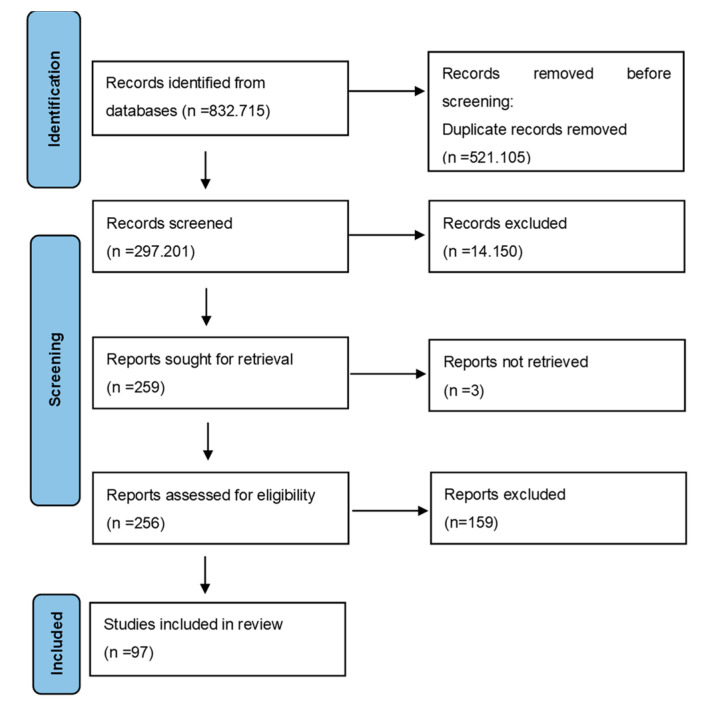
Prisma flow diagram.

Titles and abstracts were scanned by two reviewers independently (L.M.B. and L.C.R.) for possible inclusion under the above mentioned criteria. Disagreements between authors were solved through discussions and consensus and mediated by a thirdg reviewer, L.C.A. The final decision was made based on the opinion of two out of the three reviewers. The PRISMA flow chart (Figure 3) was used and guidelines were followed [31]. Studies were assessed based on the reported data.

The data extracted from each article comprised:

- Methodology used;
- Population /ethnicity;
- Sample size;
- Main conclusions;
- Accuracy of the method applied, where available.

All this information was analyzed and then tabulated in order to depict the results in a clearer manner, as the types of studies, the methodologies used and the conclusions drawn varied greatly.

## 3. Results

The studies were split into different categories and tabulated accordingly. The categories are as follows:

- Odontometric methods (Table 1);
- Radiographic methods (Table 2);
- Non-radiographic methods (Table 3);
- Ancient populations studies (Table 4);
- Biochemical methods (Table 5).

The most frequently employed parameters were MCI, MD diameter of the lower canines and ICW. Out of the total of 26 studies, 18 were performed on an Indian population. Girish et al. reported the highest accuracy of sex estimation (99.8%) by measuring the BL and MD dimensions of all upper teeth [34].

The most frequently used radiographic method was OPG, followed by CT and CBCT. The highest accuracy of sex estimation was reported by Gamba et al. (95.1%), using CBCT scans for mandibular sexual dimorphism analysis [74].

To our knowledge, so far, Gowland et al.’s study is the only one addressing the sex determination from the teeth of pre-birth individuals [106].

## 4. Discussion

In the period between January 2015 and July 2022, a large number of studies have dealt with the issue of sex estimation of individuals from measurements or analyses of the teeth and cranium, which shows the importance of the subject.

### 4.1. Populations

The most studies by far were undertaken by Indian researchers on contemporary populations, as shown in Table 1, Table 2 and Table 3 [17,18,24,33,44,61,63,66,68,70,71,72,75,76,77,78,80,81,84,89,90,91,96]. With regard to European populations, Greek studies seem to be more frequent [10,93,94,99], but there are also British [11,12], Portuguese [52,53], Spanish [54], Croatian [4,56], Bosnian [16], Italian [62] and Czech [97,99] studies, a study concerning Caucasians in general [19] and one concerning South Africans of European descent [95]. A number of articles concerned Saudi Arabian, Egyptian, Malaysian, Chinese, Korean, Jordanian, Nepalese, Iranian, Japanese, Thai, Turkish, Brazilian, Peruvian and Australian populations [4,8,9,14,43,55,57,59,60,67,69,73,74,82,83,85,86,88,92,98]. One study described 1097 autopsy cases of Caucasian, Mongoloid and Negroid individuals [87]. The type of population on which morphometric studies have been conducted is important, as the results are largely population-related and not applicable to other ethnicities. This does not apply to biochemical studies, however, where the conclusions are unrelated to the ethnicity of the individuals involved.

### 4.2. Sample Size

A few articles stand out, due the large samples involved, having over 500 cases and, in some, as many as 1296 [34,44,59,87]. Girish et al.’s odontometric study comprised 500 cast measurements—half male, half female—and their ability to differentiate gender in the population using stepwise discriminant functions was found to be very high, with 99.8% accuracy [34]. Govindaram et al.’s study is the only study reviewed that involved the measurement of roots of permanent teeth in order to find sexual dimorphism. It also had a large sample of 1000 cases, with only patients with the past three generations living in Tamil Nadu and Tamil mother tongue accepted for study. The study found a number of roots displaying sexual dimorphism, while the upper and lower canines were the most dimorphic [44]. De Boer et al. used a sample of 1097 autopsy cases with multiple ancestral origins belonging to Caucasian, Negroid and Mongoloid races, for which cranial vault thickness was measured. Differences were found between males and females, with females apparently having larger frontal cranial thickness, but the conclusion drawn was that cranial vault thickness “cannot be used as a proxy for configuring the anthropological biological profile” [87].

### 4.3. Sex Estimation in Children

Perez et al.’s article was the first study attempting to use Rickett’s PA cephalometric analysis to establish the sex of an individual of a Peruvian population. Apart from being the first study to use this type of PA analysis, its strength resides in the fact that the sample size was large (1296 cases) and also involved children (5–44 year old), which is rare in this type of study (Table 1, Table 2 and Table 3). However, their accuracy rate was between 63–75% and they concluded that Rickett’s PA cephalometric analysis is not adequate for sex determination [59].

Other studies that included children or children’s skulls include those of Singh et al., Rajkumari et al., Poongodi et al., Noble et al. and Mustafa et al. [39,65,66,92,107]. Singh’s research was performed on 500 dental casts belonging to 250 boys and 250 girls aged 3 to 5 and found significant differences between the dimensions of temporary teeth in girls and boys, with boys having larger tooth dimensions than girls [39]. This was the single odontometric study on temporary teeth that met our search criteria. Another study involving children is that of Rajkumari et al., which aimed to find sexual dimorphism by analyzing mandibular dimensions with OPG. It included the OPGs of 150 patients aged 3 to 70 years and the measurements performed were: maximum ramus width (MaxRW), minimum ramus width (MinRW), condylar height (ConH), coronoid height (CorH), projective ramus height (PH) and gonial angle (GA), recorded bilaterally. They found that MaxRW (R/L), ConH (R/L), CorH (R/L), PH (R/L) and GA (R/L) showed highly statistically significant differences between the genders [65]. Poongodi et al. also included OPGs of both children and adults (ages 4–75) in their research and the results showed significant variables in the GA and the height of the ramus [66]. Mustafa et al. searched for sexual dimorphism in the palatal arch and in the size of the incisive papillae measured from 150 dental casts of Jordanian children and reported significant size differences both in the palatal arch and the incisive papillae in children [92].

Noble et al.’s research used multidetector computed tomography (MDCT) to scan 152 juvenile crania of a Western Australian population. They acquired fifty-two 3D landmarks that were analyzed using Procrustean geometric morphometrics and found little quantifiable sexual dimorphism in individuals younger than 12 years of age, whereas, in older individuals, at 18 years of age, the prediction accuracy rates are as high as 94%, and the authors concluded that simple, linear interlandmark distances of crania could be an option for preliminary classification of skeletal remains [107].

Ziganshin et al. used liquid chromatography and mass spectrometry to analyze tooth enamel peptides from 15 deciduous teeth from fossil remains. A specific peptide containing phosphorylated Ser66 residue was found only in the enamel from deciduous teeth, suggesting its role in the enamel formation of deciduous teeth [105].

Gowland et al.’s study addressed sex determination from the teeth of nonadult human remains, including pre-birth individuals, using dimorphic enamel peptide analysis [106].

### 4.4. Odontometric Studies

**Figure 4:**
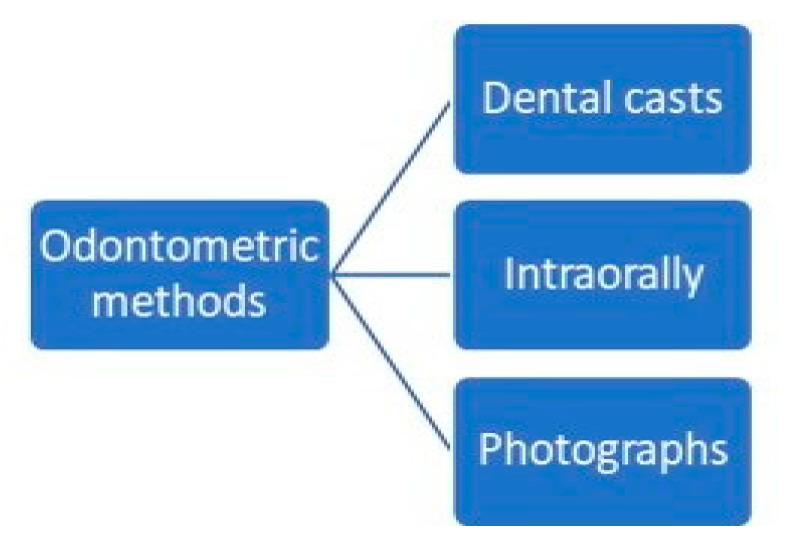
Types of odontometric methods.

Odontometric studies searched for sexual dimorphism in teeth dimensions, whether measuring upper or lower teeth, all teeth or only specific teeth. The measurements were performed intraorally [37,40,43]; from dental casts [36,38,54,55,57] or, in some cases, radiographs [40,41,57]; or using Raman spectroscopy [56], and their conclusions vary greatly in terms of the accuracy rate found (Figure 4).

The canines, maxillary central incisors and first molars (both upper and lower) [43,46] were the teeth most frequently measured and, among them, the mandibular canines seem to come up the most [40,41,45,55]. The mesiodistal and buccolingual diameters of the teeth were also frequently assessed parameters, as was the Mandibular Canine Index (MCI) [35,36,48,54,55,57].

#### 4.4.1. Mandibular Canine Index

Regarding the MCI, the results reported are very different. While Priyadharshini et al., Krishnan et al. and Silva et al. found that the MCI was not particularly useful in sex determination (Silva et al. found an accuracy rate of 64.2%) [35,50,53], other studies seem to disagree and show quite high accuracy rates, between 66.98% and 78.8%, in determining the sex by MCI [45,47,48].

#### 4.4.2. Other Teeth Measurements

Studies conducted on 28 teeth have also come up with different results. Alam et al.’s cross-sectional CBCT study performed on 159 males and 93 females of Saudi, Jordanian and Egyptian origin found that the odontometrics of the second maxillary and mandibular molars were insignificant in terms of sex estimation [42]. However, the study conducted by Girish et al. on 250 males and 250 females of Indian ancestry concluded that the ability to differentiate gender in the population using stepwise discriminant functions was very high, with 99.8% accuracy, with males showing statistically larger teeth than females [34].

Larger dimensions of teeth in males were found in Dash et al.’s study as well. They measured the MD and BL dimensions of all teeth, excluding the third molars, in an Indian population [37]. Similar to Priyadharshini et al., Krishnan et al. and Silva et al., they also concluded that canines and premolars showed no statistical difference between sexes [35,50,53].

Gouveia et al.’s research stands out from the odontometric studies through their methods. They employed experimentally burned teeth (at 400 °C, 700 °C and 900 °C) to perform measurements and test the sexual dimorphism. However, they conclude that most of the standard measurements, although presenting significant sex differences, were ”not reliable enough to allow for correct sex classifications close to 100% both before and after the burning”, but they managed to achieve correct sex classification above 80% [52].

### 4.5. Morphometrics of the Skull

**Figure 5:**
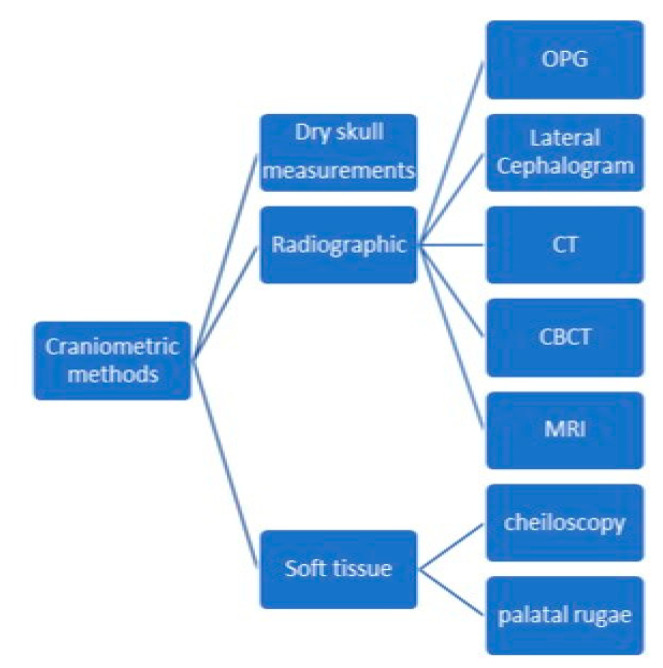
Types of craniometric methods.

Articles using morphometrics of the skull in various forms, whether through direct measurements of the skull, through radiological scans or using 3D facial computed applications, are quite difficult to compare because the methods vary greatly (Figure 5) and their conclusions are also very different.

Among the parts of the cranium most frequently assessed, studies concerning the mandible are the most frequent. Eight articles using OPG scans of the mandible, two articles using mandibular CBCT measurements, two articles using lateral cephalogram to measure mandibular parameters [78,84] and two articles employing CT (one to assess the chin and the mandibular symphysis [19] and one the mandible surface [85]) were reviewed. The most frequently measured parameters were GA and ramus height (RH).

#### 4.5.1. Dimorphism of the Gonial Angle

With regard to GA, Sambhana et al., in an OPG based study on a South Indian population, concluded that the GA did not show significant sexual dimorphism [80]. This was similar to the study by Bulut et al. [73], which examined 150 male and 150 female CT scans of the mandible of a Turkish population between the ages of 20 and 80 years old, divided into three groups for more accuracy, and concluded that the GA is not a particularly good indicator for sex identification and should not be used as a sole criterion [73]. Belaldavar et al. also found a low accuracy rate for the GA (56.3%) in their research on lateral cephalometric radiographs of 155 males and 149 females of Indian origin, aged 18–30 [78]. In contrast, Rajkumari et al., in their research on 150 OPGs, concluded that the GA, along with other mandibular parameters, such as MaxRW, ConH, CorH, and PH, showed highly statistically significant differences between the genders [65]. Similar results were found by Poongodi et al. in their OPG study, concluding that the GA and the RH are significant variables in determining the sex [66]. The study of Suzuki et al., using CT, found significant differences between Japanese males and females, the gonial angle overhanging outside in male cases [85].

#### 4.5.2. Dimorphism of the Ramus Height

RH is also often employed in morphometrics of the crania in studies performed with OPG and CBCT, with a high accuracy of prediction rates, between 69% and 83.8% [40,58,60,62,63,80,81]. With regards to this parameter, studies seem to agree more than for other parameters. Except for one study, that of Bašić et al., which only found sexual dimorphism in the mandible in its body height, the others reported high sexual dimorphism in the mandibular ramus [4]. The main difference between the study by Bašić et al. compared to all others that involved mandibular ramus measurements is that Bašić’s study was based on measurements of medieval Croatian skeletons, whereas the others were radiographic studies conducted on modern populations, most of them Indian [40,58,63,80,81] and one Saudi Arabian [60] and one Italian [62]. A particularly large sample of cases was analyzed by More et al. (500 male and 500 female digital OPGs), and the conclusion drawn was that the overall accuracy for diagnosing sex from the mandibular ramus was 69.0% [63]. Damera et al., in their study, reported that the greatest sexual dimorphism of the mandible was expressed in the maximum RH, giving an accuracy in the prediction rate of 83.8% [81]. Missier et al., in their study on 250 lateral cephalograms, reported that the RH, along with the ramus length and Conylion to Gnathion measurements, showed the highest sex-determining dependability (78%) in the mandible [58]. Similar findings were presented by Sambhana et al. in their study conducted on 384 OPGs, which resulted in an overall accuracy of 75.8%, with the CorH being the single best parameter, providing an accuracy of 74.1% [80]. The CT-based study by Suzuki et al. found significant differences regarding the size of the mandibular branch between Japanese males and females, the mandibular branch of males being larger [85].

#### 4.5.3. Dimorphism of the Chin and Mandibular Symphysis

Tunis et al.’s study regarding the chin and mandibular symphysis had a large (419) adult, age-matched sample of Caucasian origin. They concluded that males had a significantly wider and taller chin than females and, with regard to the symphysis, their study showed the existence of sexual dimorphism in the observed symphysis metric characteristics; i.e., males exhibited higher, thicker and larger symphyses that were more lingually oriented compared with those of females [19]. This was the only study reviewed concerning the chin and the mandibular symphysis.

#### 4.5.4. Dimorphism of the Foramen Magnum

Regarding the FM as a tool for sex determination, there were two types of measurements performed: area and circumference. Raikar et al. found circumference to be the best predictor of sex, achieving an accuracy rate of 67.3% [64], whereas Kamath et al.’s study found the area of the FM to be the best sex predictor [91]. Both studies were based on Indian populations, Raikar’s study being performed on 150 submentovertex radiographies while Kamath’s study was undertaken with measurements from 72 skulls.

Vinutha et al., in their research, measured the anteroposterior and transverse diameters of the FM, as well as the circumference, and 65% of cranial CT scans overall were sexed correctly based on these measurements [61].

Nourbashkh et al. performed research based on measurements of the skulls of 102 people. The frontal sinus, maxillary sinus, mandible and FM were assessed. They concluded that the highest accuracy was related to the mandible bone, with 89% (the RH had the highest value), and the lowest accuracy was related to the FM, with 71% [15].

Mahakkanukrauh et al. also measured the FM in their research, along with other measurements of dried skulls of Thai origin, and found significant differences between the genders [88].

#### 4.5.5. Dimorphism of the Maxillary Sinuses

The maxillary sinuses have also served as a tool for sex identification, but the results reported vary greatly. De Queiroz et al. measured the height and width of the maxillary sinuses and found a limited applicability for sex estimation because, when the individuals’ maxillary sinus dimensions were between certain values, it was impossible to determine the sex [67]. Rani et al.’s study was based on MRI scans of the maxillary sinuses, which was found to be an adequate method for sex estimation, with the highest sexual dimorphism being found in the volume of the left side maxillary sinus [68]. Similar results were presented by Bangi et al. in their CT study on maxillary sinuses, showing that the volume of the left maxillary sinus of males is larger than that of females [71]. Another CT-based study on maxillary sinuses was undertaken by Prabhat et al., who reported a high gender prediction accuracy of 83.3%; however, their sample size was relatively low (30 patients) [77]. In fact, except for Bangi’s research (100 cases) [71], the other reviewed studies regarding maxillary sinuses had relatively small samples: 64 cases in de Queiroz et al.’s study [67] and 60 subjects in Rani et al.’s study [68].

#### 4.5.6. Dimorphism of the Left Side versus the Right Side of the Skull

With respect to the left side of the cranium being more sexually dimorphic than the right side, Rani et al. found in their studies that the highest percentage of sexual dimorphism was shown in the left maxillary sinus [68], and similar results were reported by Bangi et al. [71]. Soman et al. also reported that the left width and area of the frontal sinus are more suitable for gender estimation [72].

#### 4.5.7. Dimorphism of the Mastoid

Regarding the mastoid, two articles were reviewed, one performed on 100 adult modern Bosnian skulls [16] and the other also performed on skulls, this time of Indian origin, all 50 adults [96]. They both concluded that the mastoid process is a good indicator for sex estimation, and the latter gave an accuracy rate for prediction of 83%. The limitation of using the mastoid process as sex estimation in forensic or anthropological investigations is related to the fact that the mastoid region is considered as one of the slowest and later-growing regions of the cranium, showing a higher degree of sexual dimorphism in adulthood, so it can only be used in adults [96].

#### 4.5.8. Dimorphism of the Palate, the Pterion and the Orbital Aperture of the Frontal Bone

Significant differences between sexes were also found in other parts of the cranium, such as the palate, pterion and orbital aperture of the frontal bone.

Two articles regarding the palate were reviewed: one performed by Mankapure et al. on 500 dental casts of adult Indian patients by measuring the arch depth and the palatal depth, which concluded that only the mean maxillary arch depth values are statistically significantly different between sexes [89]. The other study regarding the palate was undertaken by Mustafa et al. [92] on 300 dental casts, among which 150 were children. They measured the palatal arch dimensions and the size of the incisive papillae in both the adult and children groups and the shape of the incisive papillae in the adult group only. They found that the size of the palatal arch was significantly higher in adult males than females, and there were also significant differences between the size and the shape of the incisive papillae in adults. In the children group, the palatal width and length significantly predicted the sex, while the size of the incisive papillae was also significantly different between the two genders. Their conclusions strongly suggest that the palatal dimensions and their overall size are sexually dimorphic [92].

Regarding the orbital aperture, only the research done by Kanjani et al. met our search criteria. This was performed with PA cephalograms of 250 adult males and 250 adult females of North Indian origin, and the parameters measured were the maximum height and width of the right and left orbits, along with the interorbital distance. The study reported 84.8% accuracy after subjecting the obtained values to discriminant function analysis [70].

The study by Uabundit et al., carried out on 124 dried skulls, aimed to classify and examine the prevalence of all types of pterion variations using morphometric measurements and machine learning models to estimate sex and age. The main conclusion was that the random forest algorithm could predict sex with 80.7% accuracy [98]. 

### 4.6. High Sex Prediction Accuracy

Among the articles reviewed, few of them report a very high sex prediction accuracy based on morphometric or odontometric methods. Mahakkanukrauh et al.’s study, which performed various cranial measurements of the skull of 200 Thai individuals, reported that, according to discriminant analysis, percentage accuracies obtained from both direct and stepwise methods were distinctly high (88.0–92.2%) [88].

Yang et al. investigated the superior orbital margin and frontal bone of the skull in a Chinese population and proposed a technology of objective sex estimation for the skull using wavelet transforms and Fourier transforms. Their results showed that the accuracy rate for male and female sex discrimination was between 90.9% and 94.4% [14].

A very high accuracy rate was also reported by Shireen et al. in their study regarding the sexual dimorphism of the frontal sinus in a Saudi Arabian population. Their reported accuracy rates were between 67.70% and 95.90% [79]. Nuzzolese et al., in their OPG-based study on the mandible, also reported that the efficacy of cross-validated discriminant analysis indicated a high level of robust and significant classification based on their *25* chosen landmarks, with 92.5% correct overall classifications [62].

The odontometric study with the highest accuracy rate reported was that of Girish et al., performed on cast models of all upper teeth except the third molars. They measured the MD and BL dimensions of these teeth and found that the ability to differentiate gender in the population using stepwise discriminant functions had a 99.8% accuracy [34].

### 4.7. Machine Learning

Machine learning and virtual methods to assess dimorphism are, most likely, the way forward in this field. Not only are they becoming more and more accurate, but they are also less time consuming, less invasive and more cost-efficient compared to other methods [9,97,98,99]. Parts of the skull or the skull as a whole are more frequently assessed through these methods, as in the studies undertaken by Gao et al. [9], Chovalpoulou et al. [94,99], Arigbabu et al. [8], Musilova et al. [97], Uabundit et al. [98] and Bertsatos et al. [99]. However, soft tissue can also serve to determine the dimorphic features of the face, as in Agbolade et al.’s study [11]. Noble et al.’s study on juvenile crania also employed machine learning methods [107].

### 4.8. Biochemical Analysis

The biochemical methods used for sex estimation were performed either on teeth alone [23,24,101], on teeth and bone [30,103] or on bone alone [102].

Both Chowdhury et al., and Dutta et al. [23,24] performed their research on teeth subjected to different conditions mimicking environmental conditions, such as teeth buried in soil or under extreme heat, and attempted to amplify the Amel gene from dental pulp or dentin using the PCR reaction. Chowdhury et al. found that the amount of DNA extracted decreases as the period of time in which teeth were exposed increases, that teeth buried in soil yielded the least amount of DNA over a period of time and that no DNA could be obtained at high temperatures (350 °C) [23]. Dutta et al.’s research was performed on 50 teeth samples also exposed to different conditions, such as sea water, room temperature, soil and incineration (500–1050 °C) [24]. They achieved 100% retrieval of DNA along with gender determination, even under extreme environmental conditions (1050 °C), which was not reported elsewhere in the literature and gives the study particular strength. Their reported limitation lies in the high number of PCR cycles needed and in the fact that it was time-consuming in cases of salt-water exposure and incineration [24].

Both Pilli et al.’s and Gonzalez et al.’s studies compared the quality of DNA extracted from teeth to that extracted from petrous bone and their results were similar, in that both studies found that the petrous bone was the best skeletal element with regard to skeletal conservation [30,103]. Pilli et al.’s research was conducted on ancient skeletal remains from the 6th to 7th century CE and found that it was also possible to obtain a complete STR profile when analyzing ancient bones [30]. Gonzalez et al. also performed a histological analysis as well to compare the microscopic structure of a petrous bone to that of a tooth and the microscopic structure of fresh petrous bone to that of an archaeological or forensic sample, trying to understand why the petrous bone is an advantageous substrate in ancient DNA studies. They found a ”peculiar microstructural characteristic, unique to the petrous bone, that might explain the good preservation of DNA in that substrate” [103].

Kulstein et al. based their research on comparing the petrous bone to other parts of cranial bones in trying to retrieve DNA. They showed that STR typing from the petrous bones led to reportable profiles in all individuals. They also compared the efficacy of two techniques—namely, CE typing and MPS analysis—and showed that ”MPS has the potential to analyze degraded human remains and is even capable to provide additional information about phenotype and ancestry of unknown individuals” [102].

The study by Froment et al. emphasized the high potential of MS-based proteomics as an alternative for sex estimation of ancient remains when DNA is not exploitable [104].

The studies by Ziganshin et al. [105] and Gowland et al. [106] investigated the role of enamel peptides in the sex determination of human remains, with promising results.

## 5. Conclusions

Except for biochemical analysis, there is no single morphometric or morphological method reporting 100% accurate results regarding sex estimation. However, the multitude of methods tested and the continuous development of new techniques, especially computer-aided technologies and high-quality radiological images, and advances in the dental and forensic research fields have improved gender determination methods over the last years and will probably continue to do so in the future. The high volume of articles and the high number of researchers, with various backgrounds, concerned about this topic show the importance of this subject for scientists, dentists, forensic investigators and anthropologists alike.

## Figures and Tables

**Table 1:** Odontometric methods.

No.	Reference	Methodology	Population	No. of Cases/Age	Main Conclusions	Sex Estimation Accuracy
1	[32]	Linear and diagonal dimensions recorded at both crown and cementoenamel junction levels of extracted molars	Northwest Indian	73 males 57 females	The calculated index of sexual dimorphism was higher in lower molars than in the upper molars	Max 70%
2	[33]	Four odontometric parameters: ICW, IPW, AL and CW, measured directly with the subject	Indian	100 males 100 females	Maxillary parameters exhibited higher mean values in males compared to females	
3	[34]	MD and BL dimensions of all upper teeth	Indian	250 males 250 females	The MD and BL dimensions were statistically significant different between males and females	99.8% using stepwise discriminant functions
4	[35]	Lip prints; Mandibular Canine Index; Facial Index	Indian	50 males 50 females	Type II pattern in lips most common No significant difference in odontometric analysis	
5	[36]	Maxillary impressions; palatine rugae; MD canines; ICW; MD and BL of upper molars	Indian	60 males 60 females 20 families of 4 members	Females—more wavy rugaes Males—all measured indexes were higher than in females	
6	[37]	MD, BL measurements of 28 teeth	Indian	100 males 100 females 18–25 years old	Larger dimensions of teeth in males when compared to females	
7	[38]	Maxillary Canine Index and maxillary first molar dimensions	Indian	100 males 100 females 15–25 years old	BL dimension of maxillary first molar is a more reliable indicator for gender determination	
8	[39]	MD and BL dimensions of upper and lower *temporary teeth*	Indian	250 males 250 females 3–5 years old	Boys generally had larger crown diameters than girls	
9	[40]	Maximum ramus height, bigonion width and bicondylar breadth in OPG MD of upper central incisors, canines	Indian	100 males 100 females 18–30 years old	Ramus height—most dimorphic Permanent maxillary central incisor—more dimorphic than the maxillary canines	
10	[41]	MD—left mandibular canine	Indian	60 males 60 females 15–40 years old	Increased MD diameter in males	72.5%
11	[42]	CBCT and odontometrics of 28 teeth	Jordanian, Saudi, Egyptian	159 males 93 females 20–45 years old	Odontometric differences of 28 teeth between gender and among Saudi, Jordanian and Egyptian populations were insignificant (*p* > 0.05)	
12	[43]	MD and BL of permanent upper first molar	Indian	300 males 300 females 17–25 years old	The differences between males and females in MD and BL dimensions measured were statistically significant (*p* < 0.05)	
13	[44]	OPG—root length observed in all permanent teeth	Indian	500 males 500 females 21–60 years old	Sexual dimorphism in root length was observed in 13, 14, 15, 16, 23, 26, 33, 36, 43 and 46 (mesial) Most dimorphic teeth were canines	
14	[45]	MD of left and right canine, intercanine distance, MCI	Indian	100 males 100 females 18–25 years old	Significant sexual dimorphism of mandibular canines	73%
15	[46]	MD and BL of upper first molar	Indian	149 males 151 females 18–30 years old	BL crown dimension and the hypocone (distolingual) cusp showed the highest sexual dimorphism	64.3%
16	[47]	MCI and Pont Index	Indian	53 males 53 females 18–25 years old	MCI and Pont’s Index showed significant sexual dimorphism	Standard right MCI could predict sex accurately at 75.4% Standard left MCI could predict sex accurately at 66.9%
17	[48]	MD diameter of permanent mandibular right and left canines, as well as mandibular intercanine distance	Indian	200 males 200 females 20–40 years old	The MD crown width of the permanent mandibular right and left canines, as well as the mandibular intercanine distance of the males, was found to be larger in size	78.8%
18	[49]	MD and BL diameter of mandibular canine and mandibular first molar—study casts	Indian	50 males 50 females 17–25 years old	Sexual dimorphism can be predicted by measuring mesiodistal dimension of mandibular canine and mandibular first molar	
19	[50]	Lip prints Finger prints MCI	Indian	25 males 25 females 18–25 years old	MCI was not found to be a significant indicator of gender Lip prints exhibited sexual dimorphism	
20	[51]	Dental measurements on upper right teeth	Brazilian	100 males 100 females 18–30 years old	Dental measurements are useful tools for sex determination, and the canine measurements showed a proportional correlation with stature	70.5%
21	[52]	Experimentally burned teeth at 400 °C, 700 °C and 900 °C	Portuguese		The perimeter at the CEJ and the combined measurements of the MD and BL diameters, at the same level, were quite promising in the post-burning analysis	˃80%
22	[53]	MCI measured from dental casts	Portuguese	50 males 70 females 16–30 years old	MCI may not be particularly useful in sex prediction	64.2%
23	[54]	MD dimension of teeth from study castsPCA from the logarithm of the dental widths	Spanish	120 patients mean age: 14.48 ± 2.78 (males) mean age: 14.71 ± 2.69 (females)	Tooth dimension can be a considered a valuable complementary tool in sex determination for Spanish population	76.2%
24	[55]	MD widths of mandibular canines ICW From casts	Nepal	40 male 40 female	Sex predictability by using MCI showed poor sex predictability and should be used cautiously in Nepalese population	57.5–62.5%
25	[56]	Raman spectroscopy of teeth PCA of teeth from anthropological collection	Croatian	55 teeth 11–76 years old	The accuracy of classification models depends both on the tooth type (molar and premolar) and recording site (anatomical neck and apex) on the tooth	˃90%
26	[57]	MD and BL dimensions of permanent teeth measured from dental casts and radiographs	Iranian	74 male 257 female 12–35 years old	Sex dimorphism is very strong in the dentition Ageing significantly reduces measurements Mandibular canines were the most dimorphic teethBolton ratio was not affected by sex	

ICW—intercanine width; IPW—interpremolar width; AL—arch length; CW—combined length of six maxillary anterior teeth; MD—mesiodistal; BL—buccolingual; OPG—ortopanthomography; CBCT—cone-beam computed tomography; MCI—Mandibular Canine Index; CEJ—cement–enamel junction; PCA—principal component analysis.

**Table 2:** Radiographic methods.

No.	Reference	Methodology	Population	No. of Cases/Age	Main Conclusions	Sex Estimation Accuracy
1	[42]	CBCT and odontometrics of 28 teeth	Jordanian, Saudi, Egyptian	159 males 93 females, 20–45 years old	Odontometric differences of 28 teeth between gender and among Saudi, Jordanian and Egyptian populations were insignificant (*p* > 0.05)	
2	[58]	A total of 99 cephalometric variables were compared, subjected to statistical analysis and tested for significance using the *t*-test	Dravidian	125 males 125 females 25–40 years old	Twenty-four variables showed statistical significance	52—78%
3	[59]	PA cephalometric analysis	Hispano-American Peruvians	1525 patients 5–44 years old	Significant differences between sexes Males, on average, are larger and have increased muscle attachment in their skeletons than females	63–75%
4	[60]	Mandible morphometry on CBCT scans	Korean	96 males 104 females 18–60 years old	Gender can be accurately predicted using this technique	67%
5	[61]	CT scans of FM	Indian	110 males 90 females	Shape and dimensions of FM should be taken into consideration during surgery involving the craniovertebral junction and in forensic and anthropological investigations	65%
6	[62]	Morphometric analysis of the mandible with OPG	Italian	50 males 20–68 years 50 females 21–62 years old	Mandible exhibits great sexual dimorphism	92.5%
7	[63]	Morphometric analysis with OPG	Indian	500 males 500 females 21–60 years old		69%
8	[64]	Submentovertex radiography	South Indian	75 males 75 females	Circumference in FM was the best sex indicator	67.3%
9	[65]	OPG measurements of the mandible	Chennai	150 OPGs 3–70 years old, divided into seven groups	Highly statistically significant differences between genders	
10	[66]	OPG measurements of the mandible	Indian	113 males 87 females 4–75 years old	Significant differences between all the parameters: gonial angle, height and width of the ramus of mandible	
11	[67]	Maxillary sinuses measured with OPG	Brazilian	32 males 32 females ˃20 years old	There were differences between the mean values of the maxillary sinus dimensions evaluated for both sexes However, when the values were between 27 mm and 31 mm for height, and 44 mm and 48 mm for width, it was impossible to determine the sex	
12	[68]	Maxillary sinus measurements with MRI scan	Indian	30 males 30 females 21–73 years old	Sexual dimorphism was shown by the volume of the maxillary sinuses on the left side	
13	[69]	CT scans of skulls	Malaysian	45 males 42 females 18–75 years old	Males showed higher values for all the parameters than females, except for the left orbital height	85.1%
14	[70]	Orbital aperture dimension with PA cephalogram	North Indian	250 males 250 females 20–50 years old	All the linear measurements, such as orbital height, orbital width and interorbital distance, were significantly greater in males than females	84.8%
15	[71]	Maxillary sinuses measured with CT scans	Indian	50 males 50 females ˃20 years old	Volume of left maxillary sinus of males is larger than that of females	84% in males 92% in females
16	[19]	Chin and mandibular symphysis measurements with CT scans	Caucasian	203 males 216 females ˃18 years old Age-matched samples	Chin width (the frontal view) was found to be a sexually selected trait; it can be considered as a parameter for sex determination The chin was found to be a more heterogeneous anatomical structure than symphysis and it was sexually more dismorphic	
17	[72]	Frontal sinus measured with PA cephalograms	Indian	100 males 100 females ≥14 years old	It was found that the left width and area are most suitable for gender determination	
18	[73]	CT scans of the gonial angle	Turkish	150 males 150 females Three age groups 20–80 years old	Males showed slightly smaller gonial angle values than those of females in all age groups Gonial angle is not a particularly good indicator to identify the sex from the cranium	
19	[74]	Mandibular CBCT scans	Brazilian	74 males 86 females 18–60 years old		95.1%
20	[75]	Bi-zygomatic distance and intervolt distance measured with”jug handle” radiograph	Indian	30 males 30 females 18–25 years old	Bizygomatic distance is a more reliable parameter to determine gender as compared to intervault distance	
21	[17]	Mandibular ramus and gonial angle measurements with OPG	North Indian	200 males 200 females 10–40 years old	The mandibular ramus showed a high sexual dimorphism, with condylar and coronoid ramus heights as the most significant predictor for age and sex estimation Gonial angle can only be used as an additional tool	
22	[18]	Mandibular rami measurements with OPG	South Indian	229 males 271 females 20–60 years old	Condylar height/maximum ramus height was found to be the best sex predictor	80.4%
23	[15]	CBCT measurements of the skull	Iranian	51 males 51 females 46.65 ± 12.72 years old		Highest accuracy related to mandible bone—89% Lowest accuracy related to FM—71%
24	[76]	Clivus measurements with CBCT scan	Indian	76 males 74 females 6–17 years old	The clivus length was statistically significant The clivus length was greater in male population	
25	[44]	OPG—root length observed in all permanent teeth	Indian	500 males 500 females 21–60 years old	Sexual dimorphism in root length was observed in 13, 14, 15, 16, 23, 26, 33, 36, 43 and 46 (mesial); The most dimorphic teeth are canines	
26	[77]	CT images used to measure the mediolateral, superoinferior and anteroposterior dimensions and the volume of the maxillary sinuses	Indian	15 males 15 females		83.3%
27	[78]	Lateral cephalograms—gonial angle	Indian	149 males 155 females 18–30 years old		56.3%
28	[79]	Morphometric evaluation of frontal sinus with PA radiographs	Saudi Arabian	200 males 200 females 14–70 years old	Right width and left width are most suited regressors for sex determination	67.70–95.90%
29	[80]	OPG—ten mandibular variables were measured	South Indian	192 males 192 females	Coronoid height was the single best parameter, providing an accuracy of 74.1%	Overall accuracy: 75.8%
30	[81]	Measurements of the mandibular ramus: maximum ramus breadth, maximum ramus height and coronoid height using Planmeca ProMax	Indian	80 OPGs	Greatest sexual dimorphism was noticed in the maximum ramus height	Prediction rate using all five variables: 83.8%
31	[82]	Linear tooth measurements with CBCT machine learning: naive Bayesian, random forest, support vector machine	Iranian	245 males 240 females	Naive Bayesian—highest accuracy for sex classification	Average accuracy: 92.31%
32	[83]	Roof, height and floor of pulp chamber Marginal enamel/dentine thickness Tooth width and crown length CBCT	Iranian	100 males 100 females Mean age: 21.28 ± 2.47	Maxillary first molars were more dimorphic than mandibular teeth Mesio-distal variables were more dimorphic than bucco-lingual ones	Highest accuracy: 84%
33	[84]	PCA with lateral cephalograms	Indian	54 males 51 females	Sex was clearly associated with occlusion	Over 96% variation between male and female
34	[85]	PCA of mandible surface CT scans	Japanese	23 males 22 females Mean age: 43.1 ± 14.6	Significant differences between male and female, the mandibular branch of males was larger than that of females, and the mandible angle was overhanging outside	

PA—postero-anterior; FM—foramen magnum; MRI—magnetic resonance imaging; CT—computed tomography; HBM—homologous body modeling.

**Table 3:** Non-radiographic methods (cranial morphometric studies on modern populations).

No.	Reference	Methodology	Population	No. of Cases/Age	Main Conclusions	Sex Estimation Accuracy
1	[9]	Morphological features from the 3D skull MKDSIF-FCM algorithm	Han Chinese		Accuracy improvements of nearly 8.6%, 3.5% and 2.2% compared to other algorithms	
2	[14]	Supraorbital margin and frontal bone quantified by wavelet transform and Fourier transform	Han Chinese	73 males 60 females 22–28 years old	Compared with the traditional methods, the correct rate is higher	90.9% for males 94.4% for females
3	[86]	Photographs of maxillary sutures—dry skulls	Thai	96 males 94 females	Maxillary suture length can be applied for sex estimation	79.47%
4	[87]	Cranial vault thickness—autopsy cases	Caucasion Negroid Mongoloid	1097 cases 103 ˂ 19 years old 994 ˃ 19 years old	Females appear to have a larger frontal cranial thickness Cranial vault thickness generally cannot be used as an indicator for sex	
5	[88]	Various craniometric measurements on dry skulls	Thai	100 males 36–96 years old 100 females 15–93 years old	Mastoid length (right and left), nasal height, FM length, cranial base length, bizygomatic breadth, FM breadth, biauricular breadth, upper facial breadth, basion-nasospinale length, maximum cranial length and biorbital breadth expressed significant sexual dimorphism	88–92.2%
6	[89]	Maxillary arch depth and palatal depth measured from dental casts	Indian	250 males 250 females 17–25 years	Only mean maxillary arch depth values were found to be statistically significantly different	
7	[90]	Anthropometric measurements of patients	Indian	50 males 50 females 30–40 years old	Significantly higher facial height, pronasale-to-menton distance and interzygomatic width in males as compared to females	
8	[91]	Measurements of FM in skulls	Indian	41 males 31 females ˃18 years old		Predictability of area was the highest:70.3%
9	[92]	Palate measurements from dental casts	Jordanian	66 males 84 females 18–50 years old 75 males 75 females 6–12 years old	The palatal dimensions that reflect the palatal size were significantly higher in males than in females	
10	[11]	3D soft tissue craniofacial analysis	British and Irish white Europeans	102 British males 27 Irish males 132 British females 31 Irish females Below 13–over 50 years old	The magnitude of dimorphism in sex is revealed in facial, nasal and crania measurements Males are relatively larger than females, especially in the mouth and nasal regions	
11	[93]	Skull measurements	Greek	176 individuals		Multivariate combinations: ˃95%
12	[94]	Vault and midsagittal curve of the neurocranium measurements	Greek	94 males 82 females	In contrast to the midsagittal curve of the neurocranium, the shape of the cranial vault can be used as an indicator of sex in the modern Greek population	89.2%
13	[95]	Novel interlandmark distance measures across six regions of the cranium (dry skulls)	South Africans of European descent (white)	114 males 113 females		74–88.2%
14	[10]	3D geometric morphometric measurements of the cranium (dry skulls)	Greek	94 males 82 females	There are shape differences between the sexes in the upper-face and the orbits Size is significant for sexual dimorphism in the upper-face region	
15	[16]	Mastoid process measurements from dry skulls	Bosnian	50 males 47–71 year old 50 females 43–76 years old	There was a statistically significant difference between the genders on the basis of the mastoid process	
16	[96]	Mastoid measurements from dry skull	Indian	25 males 25 females ˃18 years old	The mastoid process is a good indicator for sex determination	83%
17	[12]	Mandible measurements from dry skulls	British	40 males 36 females	Mandibular metrics are good predictors for sex determination	77.3%
18	[8]	Computer vision cranial measurements	Malaysian	54 males 46 females 5–85 years old	CV methods are suitable for sex determination	78.2–86.2%
19	[97]	Virtual method—evaluating the exocranial surface	Czech	208 individuals	Highest accuracy for Czech population—96.2% Highest accuracy for inter-populational differences—92.8%	91.8%
20	[98]	Pterion surface evaluated by machine learning	Thai	100 skulls	PMP and PI distances were significantly longer in males	80.7%
21	[99]	Fully automated method with 3D models	CzechGreek	170 Czech 156 Greek	The method is efficient in estimating sex from cranial remains	Population-specific accuracy: 78.5–96.7% Population generic accuracy: 71.7–90.8%

PMP—distance from the center of the pterion to the mastoid process of the temporal bone; PI—distance from the center of the pterion to the mastoid process of the external occipital protuberance.

**Table 4:** Ancient population studies.

No.	Reference	Methodology	Population	No. of Cases/Age	Main Conclusions	Sex Estimation Accuracy
1	[4]	Various anthropological procedures of the skull and skeleton aDNA analysis	Croatian	84 adult medievalskeletons	For the mandible, the only measurement that showed sexual dimorphism was mandibular body height	Seven multivariate and five univariate discriminant functions for sex estimation with overall accuracy rates above 80%
2	[100]	Os coxae Skull Os coxae + skull		66 individuals 13–16th century	The preauricular sulcus, frontal bossing and arc compose should be reconsidered as appropriate traits for sex estimation	The combined estimate (97.7%) outperformed the os coxae-only estimate (95.7%), which outperformed the skull-only estimate (90.4%)

**Table 5:** Biochemical studies.

No.	Reference	Methodology	No. of Cases	Main Conclusions	Sex Estimation Accuracy
1	[23]	PCR analysis from dental pulp Amelogenin gene analysis Teeth subjected to different conditions	130 teeth	Teeth buried in soil yielded least amount of DNA over a period of time and no DNA could be obtained at high temperatures	
2	[101]	PCR analysis	Eight mesiodens teeth	Sex identification through DNA was possible in six out of eight cases	
3	[24]	DNA—amelogenin analysis	50 teeth subjected to different conditions, including extreme temperatures of 1050 °C	Pulpal tissue and degenerating odontoblastic processes provided enough DNA for sex identification	100% retrieval of DNA along with gender determination
4	[30]	DNA analysis of ancient petrous bone compared to femur and tooth	39 skeletal element from 13 individuals	Petrous bone is the best skeletal element with regard to DNA conservation in ancient remains	
5	[102]	Capillary electrophoresis (CE)-and massively parallel sequencing (MPS)-based analysis of petrous bone	Different sections of eight unknown cranial bones and additionally—where available—other skeletal elements	Short tandem repeat (STR) typing from the petrous bones leads to reportable profiles in all individuals	
6	[103]	DNA extraction from petrous bone and tooth	50 skeletal remains	More likely to obtain a complete STR profile from petrous bone material	
7	[104]	MS proteomics on 5000 year old teeth	11 Neolitic human teeth	The method represents an alternative for sex estimation when DNA is not exploaitable	The targeted proteomics assay allowed the confirmation of the sex in all the samples
8	[105]	Enamel peptide analysis by liquid chromatography and mass spectrometry without destruction of analyzed teeth	8 permanent, 15 deciduous teeth from fossil remains	Analysis of teeth enamel peptidome is sutable for sex determination of human fossil remains	
9	[106]	Enamel peptides	43 teeth from 29 nonadult individuals 40 gestational weeks to 19 years old from archaeological sites in England	The method enables forensic identification of nonadult human remains, including perinates	28 out of 29 individuals were identified

## Data Availability

Not applicable.
